# Vagus Nerve Stimulation as a Treatment for Catatonia: A Hypothesis

**DOI:** 10.3389/fpsyt.2019.00086

**Published:** 2019-02-27

**Authors:** David Zilles

**Affiliations:** Department of Psychiatry and Psychotherapy, University Medical Center Göttingen, Göttingen, Germany

**Keywords:** Vagus nerve stimulation, VNS, catatonia, treatment, electroconvulsive therapy, ECT

## Abstract

**Background:** Catatonia is a syndrome comprising psychomotor, behavioral, and autonomous symptoms which may occur in the context of severe schizophrenic, affective, and other mental disorders or medical conditions. Treatment options include high dose benzodiazepines (lorazepam) and electroconvulsive therapy (ECT) with some evidence for the effectiveness of glutamate antagonists. However, due to a lack of randomized controlled studies in this severely ill population, evidence base is weak.

**Methods:** On occasion of the case of a patient with treatment resistant catatonia in schizoaffective disorder, we developed the hypothesis of vagus nerve stimulation (VNS) being a potential therapy for treatment resistant catatonia.

**Results:** Based on a selective literature search, we found a remarkable overlap of the pathophysiology of catatonia on the one hand and the putative mechanisms of action of VNS on the other hand in several domains: functional brain imaging, involved neurotransmitter systems, clinical, and theoretical. We thus decided to use VNS as a single subject clinical trial. During the 1-year-follow-up, we observed a fluctuating, but ultimately marked improvement of both catatonic symptoms and general psychopathology.

**Conclusions:** We assume there is a sufficient hypothetical corroboration for the potential effectiveness of VNS as a long-term treatment in predominantly catatonic syndromes. This hypothesis could be tested in proof-of-concept clinical trials.

## Introduction

### Catatonia

Catatonia is a clinical syndrome with predominant psychomotor symptoms (e.g., stupor, mutism, negativism, stereotypies, echolalia/echopraxia), which may be accompanied by affective, behavioral, and autonomous disturbances (1, 2). According to the concept of DSM-5 and in contrast to ICD-10, catatonia may not only occur in schizophrenia but also in the context of several mental disorders as well as general medical conditions like intoxications and autoimmune encephalitis (3). Like in other psychiatric syndromes, multiple possible etiological factors may result in a relatively uniform clinical appearance. Despite this heterogeneous etiology, there are some specific treatment options. Benzodiazepines and especially intravenous or oral lorazepam seems to be highly effective and tolerable despite the high dosages required in some cases (4). In contrast to the sometimes dramatic improvement seen in acute catatonia, benzodiazepines may be less effective in chronic catatonia. Regarding the use of electroconvulsive therapy (ECT), a recent systematic review and meta-analysis found robust and consistent effects in catatonia (5). However, the authors emphasized that existing randomized controlled trials were of low quality and heterogeneous. For the glutamate antagonists amantadine and memantine, there is also some evidence from case reports suggesting their effectiveness in cases that do not respond to lorazepam and/or ECT (6, 7).

### Vagus Nerve Stimulation

Vagus nerve stimulation (VNS) is an add-on treatment for treatment resistant depression with further potential applications in other psychiatric conditions (8). A recently published, 5-year observational registry study in patients with chronic moderate to severe depression found better clinical outcome for the VNS vs. treatment-as-usual group in terms of significantly higher response and remission rates in this difficult to treat population (9). Side effects comprise hoarseness, dyspnea, throat pain, and coughing, mostly during the active stimulation intervals. Tolerability is usually well and seems to improve over time (10). Regarding the mechanism of action, most publications refer to the afferent fibers of the vagus nerve and the central projections to the nucleus tractus solitarius (11, 12) with widespread further projections influencing several neurotransmitter systems including glutamate and GABA.

## Case Report

A 46-year-old female patient diagnosed with schizoaffective disorder (this case was already described as part of a case series in Methfessel et al.(13); written informed consent for the publication of the case report was obtained from the patient's legal guardian) was transferred to our department from another psychiatric hospital where she had been treated for almost 2 years. She presented with persisting symptoms of severe psychomotor agitation, motor and verbal stereotypies, mutism, posturing, negativism, and anxiety. There were several suicide attempts in the course of her illness. As different pharmacological treatments had already failed (including lorazepam, clozapine, several other second generation antipsychotics, venlafaxine, valproic acid), we established ECT and the patient showed a marked response after only two treatment sessions. However, the patient experienced frequent relapses, sometimes only a few hours after the last ECT session. ECT was thus given daily for 1 week and then the frequency was reduced depending on the clinical picture. Despite a weekly continuation ECT and concurrent pharmacotherapy with clozapine, lorazepam, and venlafaxine, we were not able to achieve a sustained response and discharge from hospital was not possible. In this situation, we decided to offer VNS to the patient and her legal guardian as an individual clinical trial. The rationale of this (to the best of our knowledge) first-ever treatment trial of VNS in catatonia is described below. The VNS device was activated 1 day after implantation and over the next few weeks the following settings were established: output current 2.0 mA, pulse width 250 μs, signal frequency 20 Hz, on- time 14 s, off-time 0.5 min. Consistent with the known latency of the clinical effect in major depressive disorder, we observed a gradual but marked improvement of the patient's symptoms during the next 4 months (Clinical Global Impression Scale, global improvement: 2 [much improved], efficacy index: 2 [decided improvement, partial remission of symptoms]). By this time, the patient was discharged from hospital and remained stable for a few months with a nearly complete remission of catatonic symptoms. For the time being we observed a fluctuating course with temporary reoccurrence of catatonic symptoms, mostly in the context of psychosocial stress. Due to the duration and severity of her illness episode and the frequent relapses she had experienced, an intense maintenance therapy was established including VNS, weekly maintenance ECT and pharmacotherapy.

## Hypothesis

The pivotal stimulus for the idea to use VNS in treatment-resistant catatonic patients came from clinical experiences in patients with major depression. If these patients respond to ECT but experience early and frequent relapses, this might constitute an indication for additional VNS therapy. The study by Aaronsen et al. (9) found numerically higher VNS response rates in patients that previously responded to ECT, thus response to ECT might be a predictor of subsequent response to VNS. Together with the general effectiveness of both ECT and (albeit to a lesser degree) VNS in treatment resistant depression, this may lead to the hypothesis that these largely different stimulation techniques might share a common mechanism of action. Besides the antidepressant effect, most obviously both ECT (14) and VNS have anticonvulsive properties. This might be relevant as some case series well described positive effects of anticonvulsants in catatonic syndromes that did not respond to other measures (7, 15). However, the mechanism of action of VNS might—similar to ECT—be rather unspecific due to the variety of affected downstream brain regions and circuits (11, 16). Thus, also other neuropsychiatric disorders (17) presenting with catatonic symptoms might benefit from VNS.

Proceeding from these clinical considerations, we searched the scientific literature to identify possible overlaps between the pathophysiology of catatonia and putative mechanisms of action of VNS. Findings from different domains, especially brain imaging and involved neurotransmitter systems, confirm such an overlap and corroborate the potential utility of VNS in treatment resistant catatonia. For reasons of clarity, the corresponding findings will be depicted in Table 1. Important to note, we are not aiming to give a comprehensive review of all findings in the respective areas but focus on potential links between catatonia and VNS.

**Table 1:** Outline of the evidence in support of the effectiveness of VNS in catatonia.

**Domain**	**Pathophysiology of catatonia**	**Effects of VNS/mechanism of action**
Brain Imaging	• Orbitofrontal cortex (OFC) dysfunction: Alterations in OFC activation and functional connectivity during emotional stimulation may represent a trait marker predisposing patients with schizophrenia and affective psychosis to develop catatonic symptoms (18). • Catatonic patients showed higher signal decreases in the OFC during negative emotional stimulation after administration of lorazepam when compared to healthy controls (19).	• Active VNS induced a significant decrease in regional cerebral blood flow in bilateral OFC using [^15^O] H_2_O positron emission tomography (20). • Response to VNS in treatment resistant major depression was (among others) predicted by higher OFC glucose metabolism at baseline (21). • Wostyn et al. (22) performed EEG recordings in patients with epilepsy and VNS. Using source localization and connectivity analyses, OFC activity was found to be different in VNS responders vs. nonresponders depending on VNS activation (“on” vs. “off”).
Neurotransmitter Systems	• Clinical effectiveness of pharmacological therapy with benzodiazepines and especially lorazepam (7). • High prevalence of catatonia in patients with anti-N-methyl-d-aspartate (NMDA) receptor encephalitis (23). • Case reports suggest effectiveness of glutamate antagonists amantadine and memantine (6, 7).	• GABA: Effect of VNS in epilepsy could be mediated by modulation of GABA networks (24). Cortical GABA_A_ receptor density was normalized in patients with partial epilepsy one year after VNS implantation (25). • Afferent fibers of the vagus nerve project primarily to the Nucleus Tractus Solitarius (NST). VNS increases activity within the NTS. Increased GABA and reduced glutamate signaling in the NST reduces susceptibility of induced seizures (16). • There is some experimental evidence for the role of the vagus nerve in regulating glutamate excitotoxicity in ischemic stroke (11).
Clinical	See running text.	
Theoretical	According to the vagal nerve model of catatonia (26), catatonia can be seen as an innate reflex in presence of massive physical or psychological, internal, or external threats. This refers to the Polyvagal Theory (27) which posits 3 phylogenetically distinct autonomic subsystems which are linked to different behavioral functions, i.e., social communication (via facial expression, vocalization), mobilization (fight-flight behaviors) and immobilization (tonic immobility, behavioral shutdown). As all these functions may be concerned in catatonia, dysfunction of the underlying vagal nerve subsystems might cause or at least contribute to catatonic symptoms. Similarly, Moskowitz (28) primarily describes catatonia as an inadequate fear response mediated by vagal activity. This pathophysiological pathway could be modified by VNS.	

## Conclusions

Starting from a difficult-to-treat clinical case with treatment resistant catatonia and schizoaffective disorder, a hypothesis was developed postulating the potential effectiveness of VNS in catatonic syndromes. A selective literature search detected overlaps between the pathophysiology of catatonia on the one hand and the putative mechanisms of action of VNS on the other hand. Several lines of evidence corroborate the theoretical utility of VNS in catatonia. Findings from different domains, i.e., brain imaging, neurotransmitter systems, pharmacology, clinical, and theoretical considerations, ultimately led to the first-ever treatment trial of VNS in catatonia. Ultimately, a marked improvement of the patient's catatonic symptoms and general psychopathology could be observed and was followed by a fluctuating course after discharge from hospital with further need for intense and adapted maintenance treatment including ongoing VNS, frequent maintenance ECT and pharmacotherapy alongside general psychiatric and psychological care. However, both the clinical course in our patient, and the theoretical underpinnings of the hypothesis presented above are deemed sufficiently robust to justify further research on the role of VNS in catatonia. This hypothesis could be tested in proof-of-concept clinical trials. In doing so, both the obvious clinical heterogeneity and the presumably heterogeneous pathogenesis of catatonic syndromes will have to be considered as VNS might well be a treatment option for specific subgroups of patients with catatonia. These populations would have to be refined using clinical and/or biological markers as reliable predictors of VNS response. Due to the problems in recruiting these mostly severely affected patients, this will only be possible in larger multicenter studies.

## Author Contributions

DZ developed the hypothesis and wrote the manuscript.

### Conflict of Interest Statement

The author declares that the research was conducted in the absence of any commercial or financial relationships that could be construed as a potential conflict of interest. The reviewer RCW declared a past co-authorship with the author DZ to the handling editor.
